# Modeling the Impact of Climate and Landscape on the Efficacy of White Tailed Deer Vaccination for Cattle Tick Control in Northeastern Mexico

**DOI:** 10.1371/journal.pone.0102905

**Published:** 2014-07-21

**Authors:** Agustín Estrada-Peña, Diana Carreón, Consuelo Almazán, José de la Fuente

**Affiliations:** 1 Department of Parasitology, Faculty of Veterinary Medicine, Zaragoza, Spain; 2 Instituto de Ecología Aplicada, Universidad Autónoma de Tamaulipas, Ciudad Victoria, Tamaulipas, Mexico; 3 Facultad de Medicina Veterinaria y Zootecnia, Universidad Autónoma de Tamaulipas, Ciudad Victoria, Tamaulipas, Mexico; 4 SaBio, Instituto de Investigación en Recursos Cinegéticos IREC-CSIC-UCLM-JCCM, Ciudad Real, Spain; 5 Department of Veterinary Pathobiology, Center for Veterinary Health Sciences, Oklahoma State University, Stillwater, Oklahoma, United States of America; Kansas State University, United States of America

## Abstract

Cattle ticks are distributed worldwide and affect animal health and livestock production. White tailed deer (WTD) sustain and spread cattle tick populations. The aim of this study was to model the efficacy of anti-tick vaccination of WTD to control tick infestations in the absence of cattle vaccination in a territory where both host species coexist and sustain cattle tick populations. Agent-based models that included land cover/landscape properties (patch size, distances to patches) and climatic conditions were built in a GIS environment to simulate WTD vaccine effectiveness under conditions where unvaccinated cattle shared the landscape. Published and validated information on tick life cycle was used to build models describing tick mortality and developmental rates. Data from simulations were applied to a large territory in northeastern Mexico where cattle ticks are endemic and WTD and cattle share substantial portions of the habitat. WTD movements were simulated together with tick population dynamics considering the actual landscape and climatic features. The size of the vegetation patches and the distance between patches were critical for the successful control of tick infestations after WTD vaccination. The presence of well-connected, large vegetation patches proved essential for tick control, since the tick could persist in areas of highly fragmented habitat. The continued application of one yearly vaccination on days 1-70 for three years reduced tick abundance/animal/patch by a factor of 40 and 60 for *R. annulatus* and *R. microplus*, respectively when compared to non-vaccinated controls. The study showed that vaccination of WTD alone during three consecutive years could result in the reduction of cattle tick populations in northeastern Mexico. Furthermore, the results of the simulations suggested the possibility of using vaccines to prevent the spread and thus the re-introduction of cattle ticks into tick-free areas.

## Introduction

Cattle ticks, *Rhipicephalus (Boophilus) annulatus* and *R. (B.) microplus*, are a serious threat to animal health and production in many regions of the Americas, Asia, Oceania and Africa. They are also recognized as an emerging threat to the cattle industry in the United States [1]–[2]. *Rhipicephalus microplus* originated in Asia before it was introduced and has spread into much of Central and South America, as well as Mexico and southern USA [3]. *Rhipicephalus annulatus* is native to the Mediterranean basin, Near and Middle East [4].

Empirical comparative studies on tick burden, reproductive efficiency and larval hatching in samples collected from cattle and deer confirmed the role of wild ungulates in maintaining tick populations in wide areas of northeastern Mexico [2]. In particular, the white tailed deer (WTD), *Odocoileus virginianus*, was demonstrated to sustain and spread cattle tick populations and are responsible for a series of *R. microplus* outbreaks in southeastern USA [5].

Currently, only two methods are available to control ticks feeding on WTD, (a) a systemic treatment involving dispersal of ivermectin-medicated corn and (b) a topical treatment using 4-poster deer treatment bait stations and 2-poster deer treatment feeder adapters, both of which passively apply active acaricide topically to deer [3]. Vaccines against cattle ticks became available in the early 1990's as a cost-effective alternative for tick control that reduced the use of acaricides and the problems associated with them such as selection of acaricide-resistant ticks, environmental contamination and contamination of animal products with pesticide residues [6]-[9]. The efficacy of WTD vaccination as a method to reduce *R. microplus* tick infestations was demonstrated using tick BM86 and SUB antigens [10]. These experiments were conducted as pen trials in animal enclosures. Field trials have not been conducted with WTD but the efficacy of the vaccine was demonstrated in red deer under field conditions in Spain [10].

Modeling the tick population dynamics is a procedure aimed to capture the action of climate on the basic parameters of tick reproductive performance and mortality [11]. Some studies have explored the importance of the landscape composition on the movement of animals through “corridors” that connect patches of suitable habitat, and therefore their impact on the abundance of ticks [12]–[13]. It is now widely accepted that animals may move through a network of vegetation patches, and that these movements are governed by simple rules depending on both the size of the patch and the distance among patches of suitable habitat [14]–[15]. These movements affect the abundance of animals at specific points of the network, thus affecting also tick abundance [12]. Simulating animal movements, host preferences for some vegetation types and the impact of climate on ticks has been demonstrated to be a suitable way to capture the basics of the phenology and abundance of ticks [16]. These models are commonly known as “agent-based models” [17] because each animal is an “object” that moves through the landscape following simple rules. Animals carry a variable tick load, thus according to their phenology the abundance of ticks can be tracked and correlated with the landscape composition.

In this study, the efficacy of anti-tick vaccination of WTD to reduce cattle tick populations was modeled in simulated landscapes with different configurations of patches (size and distance among patches) and climate on these landscape were parameterized to model their effect on tick populations. Published and validated information on tick life cycle was used to build models describing tick mortality and developmental rates [18]–[21]. The aim of the study was to identify the constraints of the habitat spatial structure on the coexistence of cattle (unvaccinated) and WTD (vaccinated) and how climate and landscape factors affect tick control rates. Landscape features can affect the number of unvaccinated cattle and vaccinated WTD sharing the same landscape because the movement rules for both hosts are different. The information gained from these simulations was applied to an actual landscape in northeastern Mexico where WTD and cattle share the same landscape. The results were used to simulate both tick population dynamics under the actual climate features of the region and animal movements between patches and assess their effects on tick control after WTD vaccination.

## Results

### Model with simulated patches

**Figure 1:**
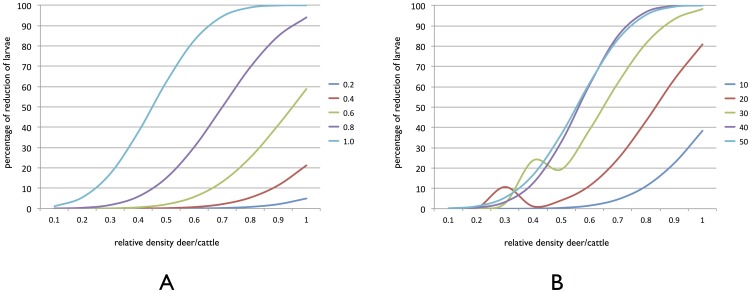
Control of tick infestations. (A) Percent reduction of tick larvae in simulated patches of vegetation, according to the host density in the patch. Each curve shows the reduction of tick larvae (in %) according to the density of vaccinated deer per hectare (colored insert). Different colored lines indicate the density of vaccinated deer per hectare (B) Percent reduction of tick larvae in simulated patches of vegetation, according to the relative host densities (unvaccinated cattle and vaccinated deer) and the size of the patch. Each curve shows the reduction of tick larvae (in %) according to the size of the patch at a fixed density of 1 deer/hectare. Different colors indicate the size of the patch in hectares.

Three critical parameters of the model had the highest impact on the reduction of the abundance of questing tick larvae, (i) absolute and relative densities of animal hosts (i.e. the actual density of each host type and their ratio), (ii) size of the patch, and (iii) degree of isolation of individual patches in the network. A fourth critical parameter was the moment of the year at which animals were vaccinated, but it was evident only when simulations were conducted using real climate data. Figure 1A shows the percent reduction of questing larvae in simulated patches of vegetation, according to the relative proportions of WTD and cattle and the density of animals per patch with vaccinated WTD only. At low densities of WTD and/or WTD/cattle ratios, vaccination protocols did not reduce tick larvae. Even with high relative densities of WTD, reduction of larvae was higher than 50% only when the abundance of WTD was higher than 0.5 animals/ha. The reduction of tick larvae in simulated patches according to the size of the patch and relative densities of both host species is shown in Figure 1B. The control of tick infestations in relatively small patches (from 10 to 30 ha) was better at high densities of vaccinated WTD but the effect was not complete. In large patches, low densities of vaccinated WTD resulted in more than 50% control of tick populations. Differences were associated with the rules governing the movements of animals across the network of patches. Therefore, while there was an obvious relationship between the isolation of the patch (the inverse of the mean distance to every linked patch) and tick control, the most important feature was patch size. The best control of tick populations was always obtained for the largest patches that have a high traversability.

### Distribution of cattle ticks in the study area

**Figure 2:**
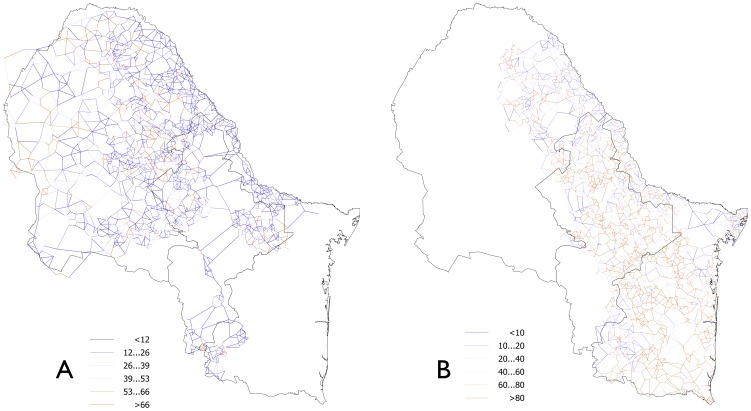
Distribution of cattle ticks in the study area. The predicted distribution of (A) *R. annulatus* and (B) *R. microplus* in northeastern Mexico. Each line shows the link between two contiguous patches forming the network of movements of hosts and ticks across the study area. The colors represent the recruitment, a value expressing the density of ticks. Values indicate higher number of ticks moving between patches.

The distribution of *R. annulatus* and *R. microplus* on WTD moving across the vegetation patches was estimated in the study area (Figure 2). After a 5 years simulation, the trends of tick abundance stabilized and remained cyclic for the entire study area. All patches larger than 2 ha were reachable by WTD and supported permanent cattle tick populations. *Rhipicephalus annulatus* was predicted to be the more abundant tick species in western parts of the study area, mainly on dried areas of northern Mexico (Figure 2). A few sites in western parts of the study area were particularly well suited for *R. annulatus* (Figure 2). The higher suitability for ticks in these areas was mainly derived from the adequate climate and improved by a network of relatively small and medium size patches located at short distances from each other. These small patches support WTD populations that contribute to the rapid dissemination of *R. annulatus*. *Rhipicephalus microplus* was predicted to be abundant in wide portions of northeastern Mexico, in sites with enough relative humidity to support low tick mortality rates (Figure 2).

### Effect of WTD vaccination on the reduction of cattle tick populations

**Figure 3:**
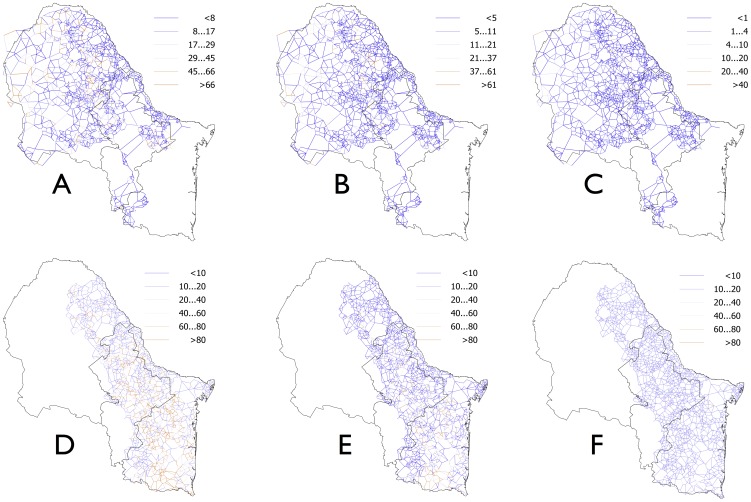
Effect of WTD vaccination on tick infestations. (A–C) Changes in the recruitment rates of *R. annulatus* in the study area each year after three years of WTD vaccination at the day 50 every year. (D–F) Changes in the recruitment rates of *R. microplus* in the study area each year after three years of WTD vaccination at the day 50 every year. Each line shows the link between two contiguous patches forming the network of movements of hosts and ticks across the study area. Different colors represent individual recruitment levels, a value indicating density of ticks. Higher values indicate higher number of ticks moving between patches.

The effect of WTD vaccination on tick populations was tested vaccinating at days 1, 70, 130, 190, 250 or 310. The simulations of tick phenology concluded that both species have variable on-host abundance peaks in the study area with two generations per year between days 126–168 and 280–322. Since vaccination schemes differed only at the moment of vaccination, they affected a different number of ticks feeding on the vaccinated WTD. The lowest tick control was obtained for protocols vaccinating on days 190, 250 and 310, because adequate protection in animals was reached well after maximum on-host tick abundance. For both tick species, vaccination around days 1 and 70 produced the best results. The continued application of one yearly vaccination on days 1–70 for three years reduced tick abundance/animal/patch by a factor of 40 and 60 for *R. annulatus* and *R. microplus*, respectively when compared to non-vaccinated controls (Figure 3). The protocol simulating vaccination on days 130 and 190 produced only a 2-fold reduction in tick abundance rates at the third year of vaccination. No significant improvements in the reduction of tick abundance rates were observed after 5 years of vaccination. An almost total lack of tick control was consistently obtained for vaccination schemes on days 250 and 310. None of the vaccination schemes completely eradicated ticks in the study area and tick pockets persisted even after three years of vaccination in sites with high habitat fragmentation, at sites where WTD are scarce or in patches small enough not to be visited by WTD.

## Discussion

Vaccination is an environmentally friendly alternative for the control of cattle tick infestations and tick-borne diseases with an impact on improving animal health and production [7]–[8], [22]–[24]. Vaccination with recombinant BM86, the antigen included in commercial vaccines for the control of cattle tick infestations [7] and SUB, a recently discovered tick protective antigen [8], demonstrated similar efficacy (76–83%) for the control of *R. microplus* infestations in WTD [10]. Therefore, these antigens were proposed as an alternative for tick control in deer [24].

Our results are the first assessment of the constraints of the landscape structure on the feasibility of the control of cattle ticks by the application of tick vaccines on WTD. Although present technology does not allow effective vaccination of wild deer, herein we showed the effect of the physical properties of the landscape and the phenology of the ticks on the outcome of vaccination protocols to control tick infestations. The model used is a special type of diffusion model [13] in which hosts must follow routes connecting patches according to some preferences based on Graph theory [14]–[15] using the evaluation of the habitat suitability for the ticks, in line with recent developments incorporating the landscape as an implicit description of tick population dynamics [16]. This basic model was able to predict the route of invasion by cattle ticks in southern Texas, before the break of the quarantine zone by infested animals was noticed [2]. In parallel with the development of models based on the climate suitability for ticks in the space (the so-called “correlative approach”), further improvements were incorporated to the description and simulation of the tick population dynamics based on the description of the processes operating at each physiological process [18], [25]. In the current development, we further addressed the explicit description of host movements by simple rules [16] and the effects derived from habitat sharing by hosts on the persistence of tick populations. This is a growing field of research [26] aimed to explain the properties of complex systems by basic rules governing the behavior of animals [27]–[28] by both locational (patch-dependent) and directional (link-dependent) rules [29]–[30].

The framework used in this research could incorporate many other features resulting in a deeper understanding of the basic processes regulating the tick seasonal dynamics. However, the inclusion of more layers of complexity aimed to improve the rules driving host movements would result in a higher uncertainty of the results, obscuring the basic relationships between ticks and hosts. It is obvious that the choice of parameters for patch occupancy and movements by animals might reshape the relationships between host movements and WTD densities at a given patch. There are not adequate censuses of WTD and cattle in the study area and thus only partial estimations of the habitat preferences for these hosts were used [30]. Because the lack of empirical data, a complete parameterization of the model has not been achieved and some facts such as the complete avoidance of some patches, the existence of geographical barriers or the restriction of the hosts because their management have not been addressed. The modeling framework allows testing these basic relationships, and shows a promising application to an actual landscape, which cannot be validated at the current stage of knowledge of field populations of ticks, cattle, and WTD.

However, several conclusions were obtained from our simulations. The control of tick populations showed close relationships with the structure of the habitat and the capture of tick phenology in the study area. These are key factors for promoting an effective tick control, because vaccination schemes targeting ticks out of the maximum on-host seasonal abundance did not work. Understanding tick population dynamics is an essential step in the evaluation of the efficacy of different control measures. Our study provided further support to the existence of a clear border dividing the suitable habitat for *R. annulatus* and *R. microplus* in the study area [2], [16], [18], [31]. This border is influenced by the deficit of water in the air as the main restrictive factor in the survival of these species of ticks. However, both tick species have a similar phenology throughout the study area. Therefore, the importance of the application of vaccination protocols at a precise time of the year, which can change from year to year because regional changes of climate, was valid for both tick species.

The second important aspect of tick ecology for control programs is tick dispersal. The impact of habitat structure on the management of cattle tick infestations by vaccination of WTD emerged in our simulated landscapes. It is known that larger patches will “attract” more animals moving on them, while shorter inter-patch distances will benefit those movements. Therefore, patches more frequently visited by the vaccinated hosts will have reduced probabilities to develop permanent tick populations. In this context, small patches closely allocated in clusters (because high fragmentation of the habitat) support frequent movements by few animals. These movements result in a higher uncertainty in the results of the vaccination. In some cases, reduction of tick population was attained but in some others the lack of habitat overlapping between WTD and cattle resulted in small pockets of remaining ticks. The driving factor of the success in tick control derives from the habitat sharing by hosts, an aspect only partially addressed in this research because the lack of empirical data. Nevertheless, WTD have a role in the maintenance and spread of *R. annulatus* and *R. microplus* populations [1], [32]–[33]. The behavior of WTD in nature has been addressed mainly regarding conservation strategies in both Texas and Mexico [34]–[35] but additional empirical studies are still necessary to understand the interactions among cattle and WTD and to define dynamic relationships of host diversity. These limitations have been recognized as a gap in our current ability to plan adequate strategies for the control of ticks and other ectoparasites affecting wild and domestic ruminants [36]. Herein, we addressed this problem by considering that WTD partially share habitat with grazing cattle, as reported for WTD in Mexico and the USA [26], [35]. However, there is evidence of a low habitat sharing between cattle and WTD in the Tamaulipas brush land [37], which occupies wide portions of the arid-steppe biome in the region, thus introducing some uncertainties in the computed data.

A recent risk assessment of potential tick and transmitted pathogens outbreaks in the temperate zone east of the South Texas Plains concluded that significant additional infrastructure and personnel would be needed to meet operational demands and treatment costs with production losses likely exceeding the economic viability of the operations under current tick elimination options [38]. It is thus necessary to evaluate the most cost-effective management strategy for tick control in the area, since the only control against the tick reintroduction into southern USA is the acaricide treatment of moving cattle, infested animals and quarantine of affected ranches in Texas [32]. However, moving of wild animals and most importantly WTD cannot be adequately managed by this strategy. Wildlife has been involved in almost all tick outbreak episodes reported in southern USA and it is targeted with the maximum priority for tick eradication in this area [2]. The results reported here suggest that WTD vaccination in northern Mexico would reduce the risk for tick outbreaks resulting in a lower risk of spreading in parts of southern USA, where movements of tick-infested WTD are common.

## Conclusions

BM86-based tick vaccines have been shown to control cattle tick populations in the field [7]. The vaccine efficacy obtained in WTD with both BM86 and SUB clearly suggests that it is possible to use these vaccines for cattle tick control in deer. The study showed that vaccinating WTD alone would reduce cattle tick populations in 3 years in northeastern Mexico. Furthermore, the results of the simulations suggest the application of these vaccines to prevent the spread and thus the re-introduction of cattle ticks into tick-free areas. These results are particularly relevant for conservation of cattle-tick free areas in the USA to prevent the possible impact of ticks and tick-borne diseases on cattle industry. Future results in the development of technologies for the application of tick vaccines to WTD under field conditions are required to apply this approach for the control of cattle tick populations in this and other regions of the world.

## Methods

### Model rationale

A model was developed to simulate population dynamics of the cattle ticks, *R. annulatus* and *R. microplus*, driven by the weather and the availability of cattle and WTD which are reported as the main hosts for these tick species in the study area [3], [39] (File S1). Artificial landscapes were produced in which patches of “habitat” were randomly allocated within a matrix of non-habitat. We used Manifold-GIS (www.manifold.net) to create random points over a background, each point being the centroid of one patch. Patches of habitat refer to sites that can be colonized by the hosts, and the model allows explicit exchange of animals following basic rules of patch size and distance among patches (see below). These patches of habitat have a range of sizes and distances among them to examine the impact of the physical features of the network on the movements of animals. Artificial landscapes of variable patch numbers and size randomly located according to a normal distribution were produced. A variable number of hosts was also distributed and randomly allocated to the complete territory at the beginning of each simulation [33], [34]. We applied the universal rule that encounters between questing ticks and hosts are governed by their abundance [40]. Since ticks quest for host “waiting” on the top of vegetation, it is assumed that high host densities will result in increased contacts between ticks and hosts [40]. Therefore, the mortality rates of the tick population while questing are lower because the effects of the climate resulting in higher tick population turnover [40]. A tick-host encounter results in the transition of the tick to the feeding stage with a certain probability of success after feeding (File S1).

Animal dispersal in a fragmented landscape depends on the complex interaction between landscape structure and animal behavior [29]. This fact has an impact on tick survival because it has been reported that tick abundance was higher in patches that are closer to a network linking the main suitable patches within the territory. Conversely, ticks were scarce or even absent within patches that were located far from the network linking the main suitable patches, even if the abiotic suitability of the patch was high [12]. These findings suggested that movements of the main tick hosts among patches support a network of tick dispersal, a hypothesis that has been confirmed by empirical data [12], [41]. The basic term of this framework is the traversability [15]. Traversability is understood as permeability of the landscape and measures the connectivity among the patches of a territory, which drives animal movements.

According to graph theory [15], the probability that an animal in node *i* will disperse to node *j* can be expressed in the form of a flux rate or dispersal probability. Such dispersal probability is directly proportional to the size of the neighboring patches, and inversely proportional to the distance to these neighboring patches (equation 1 in File S1) [15]. Total traversability is thus defined as the sum of partial dispersal probabilities for every link between two patches. The movements of the animals are governed by rules of habitat perception that relate the proportion of dispersing hosts as a response to the patch size and inter-patch distances (equation 2 in File S1) [12]. Animal movements are simulated at steps of 10 days, which is the time step used in the tick population dynamics model outlined below.

The second model component evaluates tick seasonal dynamics as driven by weather traits. The development, activity and mortality rates of the ticks were modeled according to differential equations that relate the climate features (temperature and water availability) with the physiological processes of the tick, using already published information for *R. annulatus* and *R. microplus* [18]–[21], [25]. This information was obtained from tick colonies collected in the USA-Mexico border and are therefore relevant for this study. These equations were not modified and used as published [18]–[21], [25]. Tick density is regulated by the climate-derived mortality while questing for a host, whose densities depend upon the rule of habitat perception. The relative densities of host types regulate the allocation of ticks to hosts. These differential equations were applied to each patch in the simulated landscapes to model the tick phenology and to check the changes in tick densities under different landscape configurations. All the equations used in the model development are included in File S1.

### Artificial landscapes and parameterization

Different combinations of artificial landscapes were assayed with variable size and distances of patches, and with different rules of habitat perception for both cattle and wild ungulates, the only cattle tick hosts in the study area [1]. The performance of the tick population (e.g. the production of new individuals as output of the current generation) is governed by mechanisms of density-dependent regulation according to host resistance and operating on feeding success. The models were first run in simulated landscapes to inspect for the impact of the different features of the habitat and relative densities of cattle and WTD on the tick populations.

At the beginning of the simulations, the landscape was loaded into the model together with data for the size of each patch and distance among patches (File S1). Then, the host population was allocated to the vegetation patches, with a maximum carrying capacity of 5 WTD/ha and 10 cattle/ha [39], [42]. At every decadal (10-days interval) of the year, hosts moved over the network of patches according to their physical features (size and distance; File S1). Host movements were driven by equations governing the probability that a host move to another patch according to its size and the distance between the patches (File S1). The model for tick survival and growth rates run in parallel with the host movement at 10-days intervals (File S1). Ticks find hosts depending on host density in the patch and the tick questing rates driven by climatic features. Ticks have the same preference to feed on cattle than on deer [22] and the parasitic loads on hosts depend only on host availability at the patch. At each decadal, hosts moved to another patch carrying a variable number of ticks according to the rules governing host-tick encounters. Engorged tick females dropped from the hosts and colonized patches as “visited” by the hosts. Over the time ticks were distributed over the network of patches visited by WTD or cattle. Models were run separately for *R. microplus* and *R. annulatus*. For these initial configurations, the effects of the relative WTD/cattle density were assayed from 0.1 (10% WTD, 90% WTD) to 1 (100% WTD, no cattle), different density of WTD per area of the patch (from 0.2 to 1) and the size of the patch (from 10 to 50 ha). The weather was set to optimum and constant features (22°C, 85% relative humidity) because we were parameterizing the effects of the landscape and the relative composition of hosts on the performance of the tick populations.

### WTD vaccination in simulated landscapes

Models were run for 5 consecutive years at 10-days intervals, at which every scenario produced permanent tick populations. After obtaining constant tick populations, WTD vaccination was simulated [1], [10], [22], [24]. Published data on the performance of ticks feeding on vaccinated WTD were used to simulate the effect on either *R. annulatus* or *R. microplus* [10] (File S1). For simplicity, we assumed that all hosts were adults in a stable population where changes in abundance, mortality and newborns did not occur. It was also assumed that all WTD were vaccinated in each simulation to avoid the effects of multiple levels of immunity in a large population of moving WTD.

The effect of vaccination of WTD on the tick population is thus the relationship of the animal movement in the network of patches and the number of ticks feeding on a particular host. Such feeding proportion results from the ratio between vaccinated WTD and unvaccinated cattle in each patch. We examined the importance of the patch size, the inter-patch distances and the relative abundance of each host type at patches of habitat as drivers of the percentage of tick control, measured by the reduction rate of questing larvae at each patch.

### WTD vaccination in the study area

**Figure 4:**
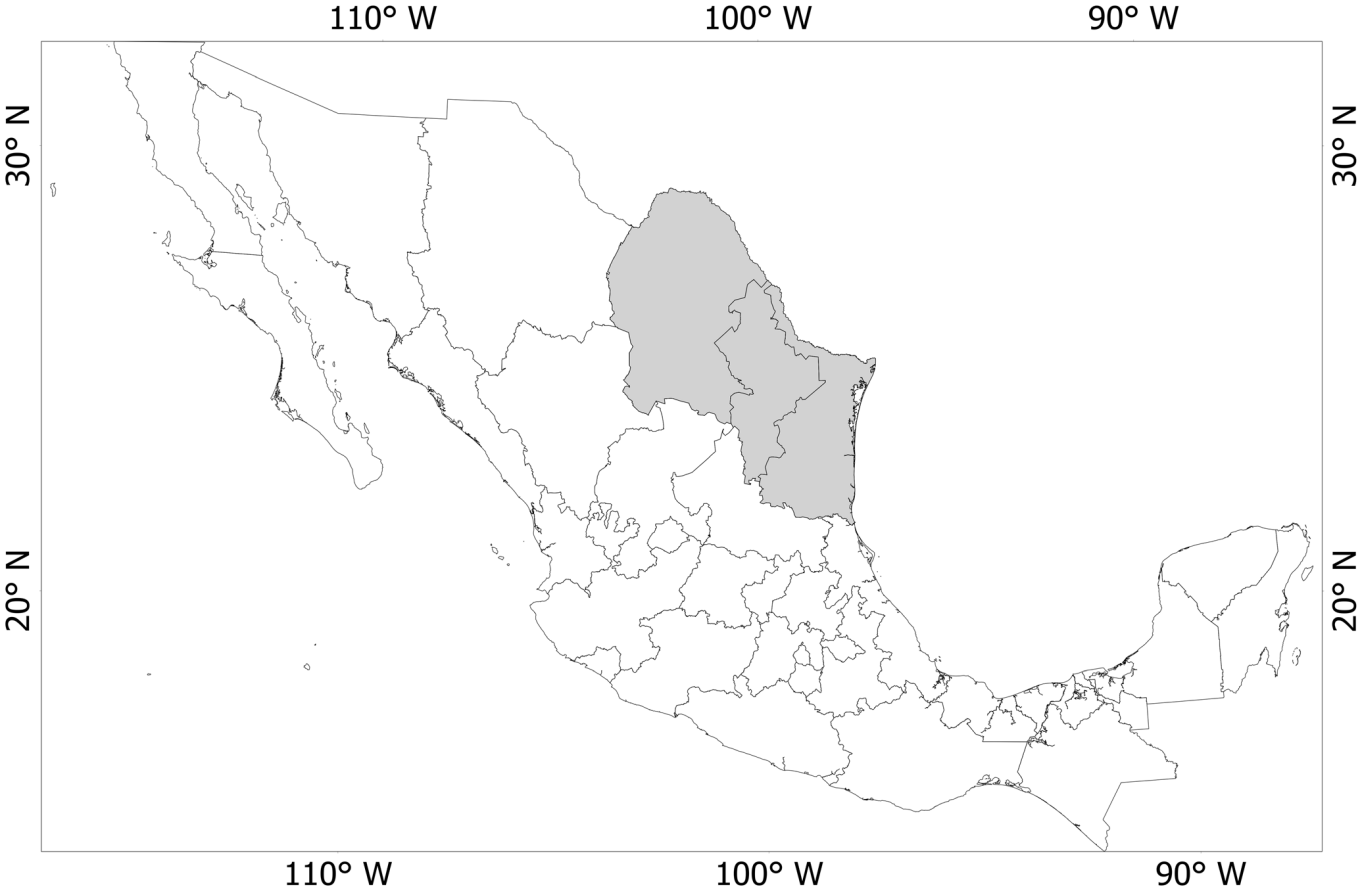
Study area. The area used for the spatial simulations of the effect of WTD vaccination to control of *R. annulatus* and *R. microplus* is marked in grey over the map of Mexico.

The knowledge gained from the simulations above was applied to a large area in northeastern Mexico. The purpose was not to evaluate a realistic vaccination protocol of WTD alone to reduce tick populations in the area because technology for mass vaccination of large WTD populations is still unavailable. We rather focused on examining how an actual landscape configuration may interfere with the actions towards tick reduction by vaccination, an intervention that must by applied in the complete territory. The study area covers a region located between 96° and 104°W and 22° and 29°N in the States of Coahuila, Nuevo León and Tamaulipas in the northeastern part of Mexico (Figure 4). Previous simulations of the landscape configuration evaluated the main spread routes of *R. microplus* from Mexico into the USA [16]. The area is thus known to have a special significance for tick control because it supports large populations of WTD [42] that has a demonstrated role in the maintenance of cattle tick populations [3], [32]. WTD in this region is believed to be responsible for the spread of tick populations into the USA [1], [11].

This part of the study was a proof of concept instead of a set of data to validate, because there is currently no way to demonstrate the findings of the model in the field. We wanted to find if an optimum strategy for WTD vaccination against both tick species exists. Tick control depends on how vaccinated WTD move through the landscape, how they share the same habitat with unvaccinated cattle, and how the seasonality of the ticks is shaped by the weather. If WTD is more abundant on critical patches of the landscape, they will carry more ticks than cattle and therefore a reduction in tick abundance will be observed. If the configuration of the landscape does not allows for a critical tick load on WTD, the effects of vaccination will be negligible.

The information used regarding the expected abundance of WTD is summarized in http://www.conabio.gob.mx/informacion/gis/?vns=gis_root/biodiv/distpot/dpmamif/dpmartio/odo_virggw (accessed on March 2012). This information was produced in the year 2009 based on published data [39] and was updated with data on WTD density obtained from 968 ranches in the study area. The WTD distribution was assumed to follow the patches of adequate vegetation and that its density is proportional to the size of each vegetation patch. The spatial distribution of vegetation patches available at the Mexican National Institute of Statistics and Geography (http://www.inegi.org.mx, accessed on March 2012) was used to calculate the carrying capacity of each patch according to its size and vegetative layer. The WTD move across the network of patches by rules governing movements according to the size of the patch of vegetation and its distance to another patches in the network. Thus, a patch must have a minimum area to be colonized; the larger the area, the more WTD will visit and stay; the shorter the distance to near patches, the less time WTD will remain at the same patch. Details about calculations of carrying capacity and movements of deer and cattle are included in File S1. Maximum carrying capacity was 5 WTD/ha and 10 cattle/ha.

In the application of the model to the study area, the impact of an annual vaccination scheme conducted for 3 consecutive years in the complete study area on days 1, 70, 130, 190, 250 and 310 was evaluated. At each decadal, animals moved through the landscape and were allocated to each patch, picking up a proportional number of ticks according to the density of hosts and ticks on each patch (File S1). Mortality of the tick females was computed for ticks feeding on vaccinated WTD and dead females were removed from the tick population. Tick fertility was then calculated for surviving females according to the immunization time and the moment of the year, and both values were introduced into the model. The seasonal dynamics of the ticks was calculated according to actual features of the weather, averaged for the period 1960–1990 and obtained from www.worldclim.org (accessed in April, 2012). This is relevant because if vaccination is carried out while only few ticks are active, the effect will be minimal.

## Supporting Information

File S1 **Description of the modeling procedures and equations governing the movements of the animals and the population dynamics of** ***R. annulatus*** **or** ***R. microplus*** **, including an R script containing the running of a general version of the model.** A schematic representation of the steps in model development and evaluation, summarizing the explanatory notes and the programming steps is also provided.
(PDF)
