# Supplementary material for: Modeling the Impact of Climate and Landscape on the Efficacy of White Tailed Deer Vaccination for Cattle Tick Control in Northeastern Mexico
Source: PLoS One. 2014 Jul 21;9(7):e102905. doi: 10.1371/journal.pone.0102905 (PMC4105637; doi:10.1371/journal.pone.0102905)
Supplement: File S1 — Description of the modeling procedures and equations governing the movements of the animals and the population dynamics of R. annulatus or R. microplus , including an R script containing the running of a general version of the model. A schematic representation of the steps in model development and evaluation, summarizing the explanatory notes and the programming steps is also provided. (PDF) [file pone.0102905.s001.pdf]

Description of modelling methods

Methods

The description of the modelling procedures adheres to the framework already proposed and developed for individual based models [1-2]. As stated in these publications, the protocols consist of three blocks that are subdivided into seven elements. Figure 1 displays the general background of the model.

Purpose

The model was developed to investigate the effect of anti-tick vaccines on wild white-tailed deer (WTD), *Odocoileus virginianus*, related to host dispersal in northeastern Mexico. Simulations of the life cycle of the cattle ticks, *R. annulatus* and *R. microplus,* was based on already published process-driven models and the effect of vaccination on tick reproductive parameters based on actual vaccine trials. The study was based on simulating a population of WTD inhabiting and moving on a 2D landscape, by using and individual based model where movement is determined by the area of each path of vegetation (carrying capacity) and the distance to near patches.

The model was not used to evaluate a realistic vaccination protocol of WTD to reduce tick infestations in the area because technology for mass vaccination of large wildlife populations is still unavailable for WTD. We rather focused on examining how an actual landscape configuration may interfere with the actions towards reduction of ticks by vaccination, an intervention that must by applied in the complete territory. The study area covers a region located between 96º and 104ºW and 22º and 29ºN in the States of Coahuila, Nuevo León and Tamaulipas in the northeastern part of Mexico (Fig. 4). Previous simulations of the landscape configuration evaluated the main spread routes of *R. microplus* from Mexico into the USA [3]. The area is thus

known to have a special significance for tick control because it supports large WTD populations [1] that has a demonstrated role in the maintenance of cattle tick populations [4]. WTD in this region are believed to be responsible for the spread of tick populations into the USA [5-6].

The objective of the study was to find the optimum strategy for WTD vaccination against both cattle tick species, which depends on how vaccinated WTD move through the landscape and how they share the same habitat with unvaccinated cattle. If WTD is more abundant on critical patches of the landscape, they will carry more ticks than cattle and therefore a reduction in the abundance of ticks will be observed. If the configuration of the landscape does not allows for a critical tick load on WTD, the effects of vaccination will be negligible. This strategy depends not only upon habitat configuration but also on the tick seasonality as driven by actual climate features.

Models were first run in simulated landscapes to assess the impact of the different features of the habitat and relative densities of cattle (unvaccinated) and WTD (vaccinated) on the control of tick populations. We then applied the model to an actual area in northeastern Mexico to examine the probable effects of vaccination on the modelled densities for the two most important tick species affecting livestock and WTD in the area, *R. microplus* and *R. annulatus*.

States variables and scales

Animal dispersal in a fragmented landscape depends on the complex interaction between landscape structure and animal behaviour. Our model uses a patch structure, in which the patches are the sites from and to where animals can move, and have variables dimensions. The tick colonization patterns strongly depend on abiotic (climate) suitability, the degree to which the patch is isolated and the overall pattern

of interconnections among patches of suitable habitat, which explains host movements and frequency [3]. Previous empirical studies [2,7-8] support the hypothesis that movements of the main tick hosts among patches support a network of tick dispersal. The movements of the WTD across the network of patches depend only upon the carrying capacity of the patch and simple rules addressing the percentage of animals that will move to another patch. Time of the year (in 10 days intervals) was used to calculate the amount of ticks active and questing for hosts. Therefore, values on tick abundance are used to calculate the amount of ticks carried by hosts in their movements.

Process overview and scheduling

A spatially explicit agent model was developed in which patches of habitat were randomly allocated within a matrix of non-habitat. The model allows the generation of patches within a range of sizes and distances among them to examine the impact of the physical features of the network on animal movements. Encounters between questing ticks and hosts are governed by mass-action. A tick-host encounter results in the transition of the tick to the feeding stage, with a certain probability of moulting success after feeding. The performance of the tick population (e.g. the production of new individuals as output of the current generation) is governed by mechanisms of density-dependent regulation, according to host resistance and acting on tick feeding success. Temperature and relative humidity act on tick survival while moulting or questing on the vegetation. Hosts were assumed to move across the landscape according to the shape and size of every patch and a set of rules involving habitat perception [9]. The density of ticks is regulated by the climate-derived mortality while questing for a host, whose densities depend on the rule of habitat perception. The relative densities of host types regulate the allocation of ticks to hosts. When host

densities were higher in a given patch, the probability of tick-host encounters increased and the tick population had a lower mortality in the questing phase and an increase in the rate in which ticks progressed from stage to stage and reproduced.

Design concepts

Emergence

Tick population dynamics are the results of the time of the year, the movements of the hosts through patches of suitable vegetation and the effect of vaccination. The time of the year has an impact because the natural phenology of the tick (as related to the weather) and the time after vaccination. Vaccination at a given period of the year will affect more or less feeding ticks on WTD. We tested the impact of an annual vaccination scheme conducted for 3 consecutive years in the complete study area on days 1, 70, 130, 190, 250 and 310. At each decadal, mortality was computed for ticks feeding on vaccinated WTD and dead females were removed from the tick population. Tick fertility was then calculated for surviving females according to the immunization time and the moment of the year, and both values were introduced into the model.

Adaptation

The adaptive traits in the model are the movements of WTD, which are driven by the carrying capacity of the patch that in turns depends upon its area and the distance to near patches in a spatial 2D network of movements.

Fitness. Reproduction of the ticks was performed according to simple rules that depend on the prevalent weather (see submodels below) and is affected by the vaccination protocols. For simplicity, we assumed that all hosts were adults in a stable population where changes in abundance, mortality and newborns did not occur. It was also assumed that all WTD were vaccinated in each simulation, to avoid the effects of

multiple levels of immunity in a large population of moving WTD.

Interaction

There is no direct interaction between individuals, but indirect interaction through density dependent foraging.

Sensing

Movement of WTD relies only on physical attributes of the vegetation patch. We used the concept of habitat use by moving hosts, carrying and spreading ticks over a simulated territory (henceforth "landscape"). Two basic terms of this framework are traversability and recruitment [10]. Traversability is understood as permeability and addresses the importance of patch network features that drive the connectivity of the territory and therefore the movements of the animals. Recruitment focuses on the estimation of tick abundance at the patch level, linking the traversability with the tick's life cycle. The recruitment of a patch is a measure of its importance within the general network and is related to the abundance of ticks in that particular patch [3].

Stochasticity

The input data are deterministic.

Observation

The key output monitored from the model was the reduction of the cattle tick, *R. annulatus* and *R. microplus*, populations.

Initialization

The model of tick life cycle was run for 5 consecutive years at 10-days intervals. After constant tick populations were obtained (e.g. each year has the same phonological component), we introduced parameters affecting tick reproductive rates by modifying the mortality rates and fertility of the feeding females on vaccinated hosts. The purpose was to simulate the effect of WTD vaccination because

vaccination with tick protective antigens results in increasing tick mortality and reduction in the fertility of the surviving female ticks after feeding on vaccinated animals [11].

The host population was allocated to the vegetation patches, with a maximum carrying capacity of 5 WTD/ha and 10 cattle/ha [19,30,35,39]. At every decade (10-days interval) of the year, hosts move over the network of patches according to their physical features (size and distance). Host movements are driven by equations governing the probability that a host move to another patch according to its size and the distance between the patches (see deer submodel below).

Input

The only external inputs were monthly temperatures and water vapor values (as saturation deficit) obtained from gridded climatology at 1 km resolution (available at http://www.climond.org). The original time resolution of the dataset (one month) was converted to 10 days interval by interpolation using splines.

Submodels

Tick submodel

We used published results to produce a process-driven model calculating the phenology and abundance of the cattle ticks, *R. annulatus* and *R. microplus,* under field conditions. All the equations presented below have been developed, validated and explained before (see references for each entry). The tick life cycle was divided into several events or processes (e.g., oviposition, molting rates, mortality) that are driven by weather conditions. All the processes are included below, together with the complete explanation of the equations and the pertinent literature data. For every equation, T is the temperature in degrees Kelvin, and SD is the saturation deficit of the air in Hectopascals.

(a) Duration of engorged female preoviposition period [12].

Rates of completion of oviposition per 10 days. Preoviposition decreases to a minimum of 2 days around 33ºC. The lower boundary is set at 12ºC [12].

$$PO\_RATE = \frac{1}{631.45 - 35.75T + 0.546T^2}$$

(b) Engorged female mortality [12].

Rates of female mortality per 10 days. A cohort of engorged females dies if it accumulates more than 83 mmHg in 10 days. To represent influence on female mortality of temperature alone, a degree-day model developed [13] was used.

FEM_MORT = (T-16)(-396.24+10.05T)

(c) Engorged female egg production (CEI: conversion efficiency index) [12].

The production of eggs by the female is a product of the temperature [14] according to the equation. This is the pure conversion of the female's body mass into eggs, and water contents of the air has not effects on it (but on the egg mortality rates, see below).

$$CEI = -159.1 + 16.63T - 0.314T^2$$

(d) Egg development rate [12,14].

To estimate the time until first hatch of an egg cohort, a function of development rate, the rate-summation embryonic development model for *R. annulatus* [15] was used. RHO25 represents the hourly development rate at 25ºC, TH the temperature at which rate-controlling enzyme becomes half active and half high-temperature inactive, TL the temperature at which rate-controlling enzyme becomes half active and half low-temperature inactive, HH the change in heat content associated with high-temperature inactivation of the enzyme, HL the change in heat content associated with low-temperature inactivation of the enzyme, HA the heat content of activation associated

with the reaction catalyzed by a rate-controlling enzyme, r the universal gas constant, T the ground temperature.

$$EGG\_DEV = \frac{RHO25(T/298.15)e^{[(HA/r)((1/298.15)-(1/T))]}}{1+e^{[(HL/r)((1/T))]}+e^{[(HH/r)((1/TH)-(1/T))]}}$$

(e) Egg mortality rate [12,14].

Mortality of eggs depends upon the saturation deficit. Egg cohorts that complete development before the mortality proportion equals 1 begin to hatch, at which time the model applies the current mortality proportion to the cohort.

(f) Duration of egg incubation [12,15].

Published data [15] showed that mean duration of egg hatch at different temperatures equaled 77% of the time required to achieve first hatch. For surviving eggs in each cohort, the model approximates temporal distribution of egg hatch with a Weibull distribution [12].

$$EGG\_HATCH = 1.005 - 0.98e^{-0.014x^{10.424}}$$

where x is the normalized development time (multiples of time to first hatch)

(g) Larval mortality rate [12,14].

Larval mortality rate is a function of temperature and water saturation deficit. Larval mortality rate is 1 in temperature < -5ºC or > 60ºC. Simulated larvae remain in the pasture until they die or attach to a host. Larval host-finding rates are explained in the next paragraph.

$$L\_DSR = 555.7 - 0.335SD$$

$$L\_TR = 555.7 - 0.335T$$

(h) Rates of contact larvae-hosts [12,14].

The rate at which questing larvae on vegetation infest cattle depends upon the

complex and highly variable process of host–parasite contact. To estimate the host-finding rate (HFR), which is the proportion of larvae picked up daily by a herd of cattle, we used equations that calculate the base host-finding rate (BHFR), a temperature-effect multiplier (TE), and a larval-density effect multiplier (LDE) [14]. The base hourly host-finding rate increases as host density (D, cows/ha or deer/ha) increases [12], according to the following formula:

$$BHFR = \frac{(0.204 * D^{0.514})}{168}$$

The multipliers used to modify this value, TE and LDE, have values bounded between 0 and 1. TE represents the increase in larval-questing activity prompted by an increase in temperature.

$$HFR = BHFR * TE * LDE$$

(i) On-host tick mortality rates [11-12].

The on-host mortality rates of either *R. microplus* or *R. annulatus* are complex relationships depending upon the age of the hosts, their immune status and the tick loads. Further complications arise because we are modeling different mortality rates produced by two different hosts (deer and cattle). We used the mortality rates previously derived and validated [14,16]. We considered the same mortality rates for ticks derived from deer based on field results [11].

Deer submodel

White tailed deer is widely distributed in Mexico [1]. We primarily used the information regarding the expected abundance of WTD in the complete country as summarised in http://www.conabio.gob.mx/informacion/gis/?vns=gis_root/biodiv/distpot/dpmamif/dpmartio/odo_virggw (accessed on March 2012). This information was produced in the

year 2009 based on published data [1]. Information about abundance and densities of WTD in the target area was updated with information obtained from 968 WTD ranches in the area of study. We assumed that WTD distribution follows the patches of adequate vegetation and that its density is proportional to the size of each vegetation patch. We used the spatial distribution of vegetation patches available at the Mexican National Institute of Statistics and Geography (http://www.inegi.org.mx, accessed on March 2012) to calculate the habitat suitability of each patch from the published information about WTD abundance, according to the size of the patch and its vegetative layer. This is the carrying capacity of each patch.

Such carrying capacity (Ccap) for each patch (i) is calculated as follows

$$Ccap_i \Big/ 1 + (Ccap_i^{1/2} * \exp(-0.2 * Area_i)$$

$$Ccap_i^j \Big/ 20 + Length_i^j$$

(a) Deer movements.

We used Manifold 8.0 software (www.manifold.net) as a standard GIS package to download the spatial information, and produce the final landscape for further modeling. The capabilities of the GIS software produced the necessary data for calculation of distances, area of patches, and rules for movements of WTD. WTD move across the network of patches by rules governing movements according to the size of a patch of vegetation and its distance to another patches in the network. Thus, a patch must have a minimum area to be colonized; the larger the area, the more WTD will visit and stay; the shorter the distance to near patches, the less time WTD will remain at the same patch. Specific routines for creation of networks are available within the software package, which produced the complete picture of the landscape with routes of movements and spatial details of every patch. To calculate the details

of the life cycle of the ticks (see below) a script in R [17] has been produced.

The host movement probability from patch *i* to patch *j* via frontier *ij* depends on the habitat perception rules. According to graph theory [10], the probability that an individual in node *i* will disperse to node *j* can be expressed in the form of a flux rate or dispersal probability matrix. Thus, the expected dispersal flux from patch *i* to *j* is:

$$f_i^j = \frac{S_i}{S_{tot}} p'^j_i$$

where *Si* is the area of patch *i*, S_{tot} is the sum of the areas of every available patch, and p'ij is the probability of dispersal from *i* to *j*. This probability of dispersal p'ij is directly related to the area of patches *i*, *j*, and inversely related to the distance between them. Total traversability is thus defined as the sum of partial dispersal flux probabilities for every link, as a measure of the permeability of that patch to propagules coming from different patches in the network of the landscape [16,18]. To define p'ij we used a function, called habitat perception, of the form:

$$p'^j_i = \frac{v}{2\pi d2\Gamma(2/v)} \exp\left[-\left(\frac{r}{d_i^j}\right)^2\right]$$

where *dij* is the distance between the centroids of patches, *r* the mean dispersal distance of the host (km) and Γ the gamma function. The parameter *v* relates the proportion of dispersing hosts as a response to the patch size. It has been assumed that proportion of moving hosts has a simple inverse relationship with patch size (i.e. small patches allow high migration rates) and the v parameter is simply a modifier of such a response in the gamma function [19].

(b) Tick questing rates, mortalities, and deer densities.

The model for tick survival and growth rates runs in parallel with the host movement

at 10-days intervals. Ticks find hosts depending on host density in the patch and the tick questing rates driven by climatic features. Therefore, the lower the density of WTD, the larger the time of quest and the higher the mortality driven by the weather and explained before in the equations for the tick submodel. Ticks have the same preference to feed on cattle than on deer [16] and the parasitic rates on hosts depend only on host availability at the patch. At each decadal, hosts move to another patch carrying a variable number of ticks according to the rules governing host-tick encounters. Engorged tick females drop from the hosts and colonize patches as "visited" by the hosts. After time, ticks are distributed over the network of patches visited by WTD and considering the effect of the climate, which describes tick development and mortality rates. Models were run separately for *R. microplus* and *R. annulatus* because their performance is different under the same climatic features [14] and results were then summarized together.

Submodel of deer vaccination

The base mortality rates were further changed after the simulation of a vaccination event. Based on published data [11] we simulated the vaccination at a given moment, by increasing the mortality and reducing the fecundity of the females fed on vaccinated deer (no vaccination is simulated on cattle). The duration of immune response on the mortality has a temporal effect, and after peaking at a maximum it decreases slowly in time [11]. The effect is also different for either *R. annulatus* or *R. microplus*. This is simulated by polynomial regressions, in which the effect of the time is evaluated in steps of 10 days. Figure 5 summarizes all the processes and submodels.

The equations for the increase of mortality for *R. microplus* (MORT_RM) and *R. annulatus* (MORT_RA) are, respectively:

$$MORT\_RM = -5.64E^{-6} \times T^4 + 0.000615 \times T^3 - 0.02225 \times T^2 + 0.267 \times T - 0.02$$

$$MORT\_RA = -4E^{-6} \times T^4 + 0.004 \times T^3 - 0.0134 \times T^2 + 0.1439 \times T + 0.2253$$

The equations relating the decrease in fecundity with the vaccination are, respectively:

$$DFEC\_RM = -0.0011E^{-6} \times T^2 + 0.04 \times T + 0.3$$

$$DFEC\_RA = -0.0014E^{-6} \times T^2 + 0.04 \times T + 0.2$$

References

1. Delfín-Alonso CA, Gallina S, López-González CA (200) Evaluación del habitat del venado cola blanca utilizando modelos espaciales y sus implicaciones para el manejo en el centro de Veracruz, México. Tropical Conservation Science 2:215-228.

2. Estrada-Peña A (2003) The relationships between habitat topology, critical scales of connectivity and tick abundance *Ixodes ricinus* in a heterogeneous landscape in northern Spain. Ecography 26: 661–671.

3. Estrada-Peña A, Venzal JM (2006) High-resolution predictive mapping for *Boophilus annulatus* and *B. microplus* (Acari: Ixodidae) in Mexico and Southern Texas. Vet Parasitol 142: 350-358.

4. George JE (1990) Wildlife as a constraint to the eradication of *Boophilus* spp. (Acari: Ixodidae). J Agric Entomol 7: 119–125.

5. Perez de Leon AA, Strickman DA, Knowles DP, Fish D, Thacker E, et al. (2010) One health approach to identify research needs in bovine and human babesioses: Workshop report. Parasites and Vectors 3: 36. http://www.parasitesandvectors.com/content/3/1/36. Accessed 15 February 2012.

6. George JE (1990) Wildlife as a constraint to the eradication of *Boophilus* spp.

(Acari: Ixodidae). J Agric Entomol 7: 119–125.

7. Tack W, Madder M, Baeten L, Vanhellemont M, Gruwez R, et al. (2012) Local habitat and landscape affect *Ixodes ricinus* tick abundances in forests on poor, sandy soils. Forest Ecol Management 265: 30–36.
8. Halos L, Bord S, Cotté V, Gasqui P, Abrial D, et al. (2010) Ecological factors characterizing the prevalence of bacterial tick-borne pathogens in *Ixodes ricinus* ticks in pastures and woodlands. Appl Environ Microbiol 76:4413-4420.
9. Vuilleumier S, Metzger R (2006) Animal dispersal modelling: handling landscape features and related animal choices. Ecol Modell 190: 159-170.
10. Urban D, Keitt T (2011) Landscape connectivity: a graph-theoretic perspective. Ecology 82: 1205-1218.
11. Carreón D, Pérez de la Lastra JM, Almazán C, Canales M, Reglero M, et al. (2012) Vaccination with BM86, subolesin and akirin protective antigens for the control of tick infestations in white tailed deer and red deer. Vaccine 30: 273-279.
12. Corson MS, Teel PD, Grant WE (2004) Microclimate influence in a physiological model of cattle-fever tick (*Boophilus* sp.) population dynamics. Ecol Modell 180: 487-514.
13. De la Vega R (1984) Aspectos del desarrollo de la garrapata del Ganado vacuno (*Boophilus microplus*) sobre el venado de cola blanca (*Odocoileus virginianus*). Revista de Salud Animal 6: 59-64.
14. Mount GA, Haile DG, Davey RB, Cooksey LM (1991) Computer simulation of *Boophilus* cattle tick (Acari: Ixodid) population dynamics. J Med Entomol 28: 223–240.

15. Strey OF, Teel PD, Ring DR, Longnecker MT (1991) Modeling embryo development and emergence of *Boophilus annulatus* (Acari: Ixodidae). J Med Entomol 28: 165-173.

16. Rodríguez-Vivas R., Ojeda-Chi MM, Rosado-Aguilar JA, Trinidad-Martínez IC, Torres-Acosta JFJ, et al. (2013) Red deer (*Cervus elaphus*) as a host for the cattle tick *Rhipicephalus microplus* (Acari: Ixodidae) in Yucatan, Mexico. *Exp Appl Acarol* DOI 10.1007/s10493-013-9672-z, accessed 2 December 2013

17. R Development Core Team (2012) R: A language and environment for statistical computing. R Foundation for Statistical Computing, Vienna, Austria, ISBN 3-900051-07-0, URL http://www.R-project.org/.

18. Hellickson MW, Campbell TA, Miller KV, Marchinton RL, DeYoung CA (2008) Seasonal ranges and site fidelity of adult male white-tailed deer (*Odocoileus virginianus*) in southern Texas. Southwestern Naturalist 53: 1-8.

19. Estrada-Peña A, Acevedo P, Ruiz-Fons F, Gortázar C, de la Fuente J (2008) Evidence of the importance of host habitat use in predicting the dilution effect of wild boar for deer exposure to *Anaplasma* spp. PLoS ONE 3 e2999. doi:10.1371/journal.pone.0002999, accessed 3 March 2012.

Figure 1. General framework for the simulations of the life cycle of *R. annulatus* and *R. microplus* on moving cattle and WTD hosts. (A) The first step of the model is loading an explicit definition of the landscape (either simulated or actual) including the size of each patch and the connecting distances between patches. (B) Animals were allocated into patches randomly. (C) Then, animals were allowed to move along patches according to simple rules involving the size of the patch, the distance between patches and the carrying capacity of each patch to produce four equations, that are averaged to obtain the probability of movements of one animal in a patch. (D) After cattle and deer populations stabilized in the network of patches, the tick submodel governs the growth rates of the tick population. (E) Vaccination started after 5 years of simulations. Two equations for both *R. annulatus* and *R. microplus* drive the mortality of the feeding instars and the fecundity of the engorged females.

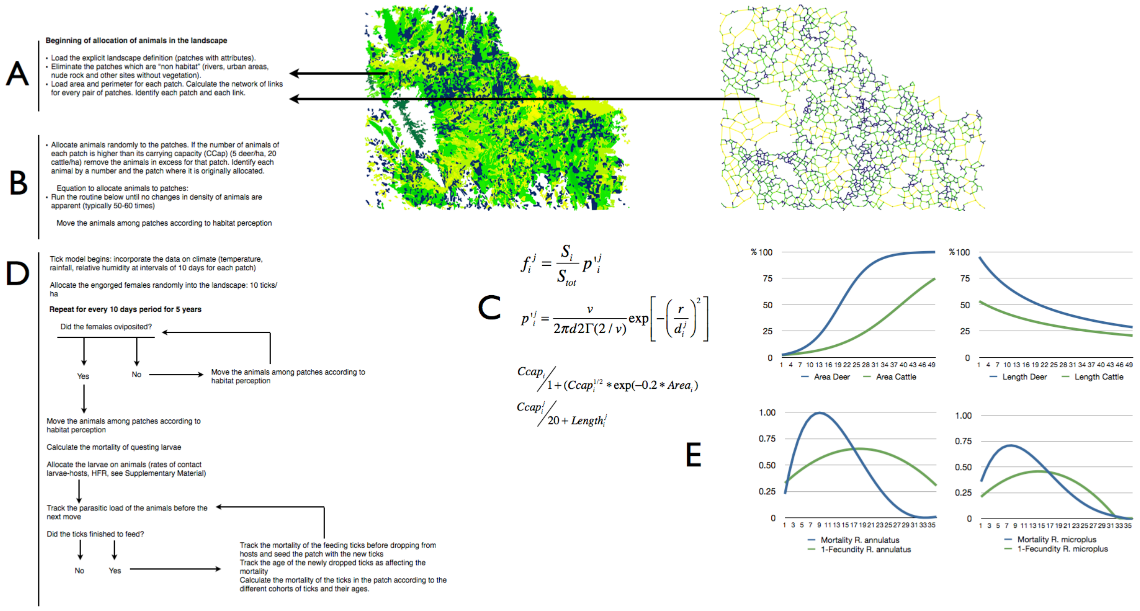

Script in R for modelling of the life cycle of *Rhipicephalus* spp. ticks

```
** Life cycle and vaccination effects on Boophilus and Deer
** version working at decadal (10 days) intervals
** code must be changed to work with other intervals
** climate data are recorded from remotely-sensed MODIS information
** landscape is explicitly considered
************************************

library(popbio)
library(maptools)
library(sp)

## Entry of files for description of landscape

puntacosTable <-
read.csv("~/Desktop/assessment_Boophilus/Boophilus/puntacosTable.csv")
CentroidsTable <-
read.csv("~/Desktop/assessment_Boophilus/Boophilus/CentroidsTable.csv")
patchesTable <-
read.csv("~/Desktop/assessment_Boophilus/Boophilus/patchesTable.csv")
pt <- readShapePoints("~/Desktop/assessment_Boophilus/puntacos.shp")

## Function definitions (life cycle)
PO_RATE <- function (i) {1/(631.45-37.57*i+0.546*i^2)}
FEM_MORT <- function (i) {(i-16)*(-396.24+10.05*i)}
CEI <- function (i) {-159.1+16.63*i-0.314*i^2}
EGG_DEV <- function (i,j,k) {RHO25*(i/298.15)*(e^(HA/j))*((1/298.15)-
(1/i))/(1+e^(HL/k))*(1/i)+e^((HH/j)*(1/TH))-(1/i)}
EGG_MORT <- function (i) {0.169+(0.000086*i)-(0.000000009*i^2)}
EGG_HATCH <- function (i) {1.005-(0.98*e^(-0.014*(i^10.424)))}
L_DSR <- function (i) {555.7-0.335*i}
L_TR <- function (i) {555.7-0.335*i}
BHFR <- function (i) {(0.204*(i^0.514)/168)}
** HFR <- BHFR*TE*LDE
MORT_RM <- function (i) {(-0.000564*i^4)+(0.000615*i^3)-
(0.02225*i^2)+(0.267*i)-0.02}
MORT_RA <- function (i) {(-0.0004*i^4)+(0.0004*i^3)-(0.0134*i^2)+(0.1439*i)-
0.2253}
DFEC_RM <- function (i) {(-0.000011*i^2)+(0.04*i)+0.3}
DFEC_RA <- function (i) {(-0.000014*i^2)+(0.04*i)+0.2}

** Section to enter the climate features from interpolated information
ClimateTemp<-matrix(data=0, nc=1311, nr=nrow(puntacosTable))
ClimateDS <- matrix(data=0, nc=1311, nr=nrow(puntacosTable))
```

```
ClimatePre <- matrix(data=0, nc=1311, nr=nrow(puntacosTable))
ClimateVap <- matrix(data=0, nc=1311, nr=nrow(puntacosTable))

casilla <- 4
for (intervalo in seq(1901,2009,by=1))
{
 print(intervalo)
 # Import temperature
 x1 <- "~/Desktop/assessment_Boophilus/temp/"
 for (mes in 1:12)
 {
  x <- paste(x1,"cru_ts_3_10.1901.2009.tmp_",intervalo,"_",mes,".asc",sep="")
  gr <- readAsciiGrid(x)
  z <- overlay(gr, pt)
  datos <- as.data.frame(z)
  ClimateTemp[,casilla] <- datos[,1]/10
  casilla <- casilla+1
 }
}
casilla <- 4
for (intervalo in seq(1901,2009,by=1))
{
 print(intervalo)
 # Import water vapor
 x1 <- "~/Desktop/assessment_Boophilus/vap/"
 for (mes in 1:12)
 {
  x <- paste(x1,"cru_ts_3_10.1901.2009.vap_",intervalo,"_",mes,".asc",sep="")
  gr <- readAsciiGrid(x)
  z <- overlay(gr, pt)
  datos <- as.data.frame(z)
  ClimateVap[,casilla] <- datos[,1]/10
  casilla <- casilla+1
 }
}
casilla <- 4
for (intervalo in seq(1901,2009,by=1))
{
 print(intervalo)
 # Import rainfall
 x1 <- "~/Desktop/assessment_Boophilus/pre/"
 for (mes in 1:12)
 {
  x <- paste(x1,"cru_ts_3_10.1901.2009.pre_",intervalo,"_",mes,".asc",sep="")
  gr <- readAsciiGrid(x)
  z <- overlay(gr, pt)
  datos <- as.data.frame(z)
  ClimatePre[,casilla] <- datos[,1]/10
  casilla <- casilla+1
 }
```

```
}
# Convert water and temperature to SD
for (columna in 1:ncol(ClimateTemp))
{
  z <- which(ClimateTemp[,columna]>0)
  ClimateDS[z,columna+3] <-
(610.78*exp(ClimateTemp[z,columna+3]/(ClimateTemp[z,columna+3]+238.3)*17.2
694)/100-ClimateVap[z,columna+3])
  z <- which(ClimateTemp[,columna+3]<0)
  ClimateDS[z,columna+3] <-
(610.78*exp(ClimateTemp[z,columna+3]/(ClimateTemp[z,columna+3]+272.4)*17.2
694)/100-ClimateVap[z,columna+3])
}
for (fila in 1:nrow(puntacosTable))
{
  ClimateTemp[,1] <- puntacosTable[,1]
  ClimateTemp[,2] <- puntacosTable[,2]
  ClimateTemp[,3] <- puntacosTable[,3]
  ClimateVap[,1] <- puntacosTable[,1]
  ClimateVap[,2] <- puntacosTable[,2]
  ClimateVap[,3] <- puntacosTable[,3]
  ClimatePre[,1] <- puntacosTable[,1]
  ClimatePre[,2] <- puntacosTable[,2]
  ClimatePre[,3] <- puntacosTable[,3]
  ClimateDS[,1] <- puntacosTable[,1]
  ClimateDS[,2] <- puntacosTable[,2]
  ClimateDS[,3] <- puntacosTable[,3]
}
#### Finish the part of loading of climate data
#### Module to allocate animals

#### Then enter number of animals
deer <-  156000
cattle <- 2300000

totalarea <- 0
for (i in 1:nrow(patchesTable))
{
  totalarea <- totalarea+patchesTable[i,3]
}
for (i in 1:nrow(patchesTable))
{
  patchesTable[i,4] <- deer*100/totalarea
  patchesTable[i,5] <- cattle*100/totalarea
}
for (i in 1:nrow(patchesTable))
{
  if (patchesTable[i,4]>patchesTable[i,3]*20) patchesTable[i,4] <-
patchesTable[i,3]*20
  if (patchesTable[i,5]>patchesTable[i,3]*2) patchesTable[i,5] <- patchesTable[i,3]*2
```

```
}

#### Life cycle begins here. Vaccine treatment is applied
#### 10 days intervals are now programmed
#### The loop includes vaccination at every week in a loop

LifeTable<-matrix(data=0, nc=9, nr=36)
colnames(LifeTable)<-
c("DevFem","DevEggs","SurvEngFem","SurvEggs","ActLarvae","SurvLarvaeOff","
GuessLarvae","GessNymphs","GuessAdults")

mueveCS <- matriz(data=0,nc=4,nr=nrow(puntacosTable))
for (year in 1:10) ### This is set to 10 years of total run
{
for (bucle2 in 1:36)
{

i<-ClimateTemp[bucle2,1]; j<-ClimateVap[bucle2,1];

PO_RATE <- function (i) {1/(631.45-37.57*i+0.546*i^2)}
FEM_MORT <- function (i) {(i-16)*(-396.24+10.05*i)}
CEI <- function (i) {-159.1+16.63*i-0.314*i^2}
EGG_DEV <- function (i,j,k) {RHO25*(i/298.15)*(e^(HA/j))*((1/298.15)-
(1/i))/(1+e^(HL/k))*(1/i)+e^((HH/j)*(1/TH))-(1/i)}
EGG_MORT <- function (i) {0.169+(0.000086*i)-(0.000000009*i^2)}
EGG_HATCH <- function (i) {1.005-(0.98*e^(-0.014*(i^10.424)))}
L_DSR <- function (i) {555.7-0.335*i}
L_TR <- function (i) {555.7-0.335*i}
BHFR <- function (i) {(0.204*(i^0.514)/168)}
** HFR <- BHFR*TE*LDE
MORT_RM <- function (i) {(-0.000564*i^4)+(0.000615*i^3)-
(0.02225*i^2)+(0.267*i)-0.02}
MORT_RA <- function (i) {(-0.0004*i^4)+(0.0004*i^3)-(0.0134*i^2)+(0.1439*i)-
0.2253}
DFEC_RM <- function (i) {(-0.000011*i^2)+(0.04*i)+0.3}
DFEC_RA <- function (i) {(-0.000014*i^2)+(0.04*i)+0.2}

### Move the animals
for (patch in 1:nrow(puntacosTable)
{
 r <- 0
 v <- 1/patchesTable[patch,1] ### Inverse of the area of the patch
 for (busca in 1:nrow(puntacosTable))
 {
  r <- r+patchesTable[patch,busca]
 }
 r <- r/nrow(puntacosTable)
 mueveCS[i,1] <- ((v/2*pi*d*2*gamma(2/v))*exp(-(r/patchesTable[i,3])^2))
 patchesTable[i,4] <- patchesTable[i,4]-mueveCS[i,1]
 patchesTable[i,6] <- patchesTable[i,5]+mueveCS[i,1]
```

```
  if (patchesTable[i,4]>patchesTable[i,3]*20) patchesTable[i,4] <-
patchesTable[i,3]*20
  if (patchesTable[i,6]>patchesTable[i,3]*2) patchesTable[i,5] <- patchesTable[i,3]*2
}

LifeTable[bucle2,1] <- PO_RATE(i);
LifeTable[bucle2,2] <- FEM_MORT(i);

LifeTable[bucle2,3] <- CEI(i);
LifeTable[bucle2,4] <- EGG_DEV(i,j,k);
LifeTable[bucle2,5] <- EGG_MORT(i);
LifeTable[bucle2,6] <- EGG_HATCH(i);

LifeTable[bucle2,7] <- L_DSR(i);
LifeTable[bucle2,9] <- L_TR(i);
LifeTable[bucle2,10] <- BHFR(i);
LifeTable[bucle2,11] <- MORT_RM(i);
LifeTable[bucle2,12] <- MORT_RA(i);
LifeTable[bucle2,13] <- DFEC_RM(i,j);
LifeTable[bucle2,14] <- DFEC_RA(i,j);
### Correct mortality of females by vaccine effect
LifeTable[bucle2,2] <- LifeTable[bucle2,2]*MORT_RM ### _RA for R. annulatus
### Corrcet fecundity of ticks by vaccine effect
LifeTable[bucle2,6] <- LifeTable[bucle2,6]*DFEC_RM ### _RA for R. annulatus
}
### Remove infinite and zero combinations
LifeTable[which(ClimateTemp<6),1] <- 0
LifeTable[which(ClimateTemp<6),2] <- 0
LifeTable[which(ClimateTemp<6),3] <- 0
LifeTable[which(ClimateTemp<6),4] <- 0

LifeTable[which(LifeTable[,1]<0),1] <- 0
LifeTable[which(LifeTable[,2]<0),2] <- 0
LifeTable[which(LifeTable[,3]<0),3] <- 0
LifeTable[which(LifeTable[,4]<0),4] <- 0
LifeTable[which(LifeTable[,1]>1),1] <- 1
LifeTable[which(LifeTable[,2]>1),2] <- 1
LifeTable[which(LifeTable[,3]>1),3] <- 1
LifeTable[which(LifeTable[,4]>1),4] <- 1

LifeTable[which(LifeTable[,5]<0),5] <- 0
LifeTable[which(LifeTable[,6]<0),6] <- 0
LifeTable[which(LifeTable[,7]<0),7] <- 0
LifeTable[which(LifeTable[,8]<0),8] <- 0
LifeTable[which(LifeTable[,5]>1),5] <- 1
LifeTable[which(LifeTable[,6]>1),6] <- 1
LifeTable[which(LifeTable[,7]>1),7] <- 1
LifeTable[which(LifeTable[,8]>1),8] <- 1

LifeTable[which(LifeTable[,9]<0),9] <- 0
```

```
LifeTable[which(LifeTable[,10]<0),10] <- 0
LifeTable[which(LifeTable[,11]<0),11] <- 0

LifeTable[which(ClimateTemp<6),9] <- 0
LifeTable[which(ClimateTemp<10),10] <- 0

# Recruitment by development of adult ticks
acumula<-0; puntero<-1; volvedor<-1
repeat
{
  while (acumula<1)
    if (puntero>nrow(ClimateTemp)) break
  else {acumula<-acumula+LifeTable[puntero,4]; puntero<-puntero+1}
  if (puntero>nrow(ClimateTemp)) break
  else LifeTable[volvedor,17]<-puntero; acumula<-0; puntero<-volvedor+1;
volvedor<-volvedor+1
}
minimLarvae <- min(LifeTable[which(LifeTable[,15]>0),15])
minimNymphs <- min(LifeTable[which(LifeTable[,16]>0),16])
minimAdults <- min(LifeTable[which(LifeTable[,17]>0),17])

for (ciclo in 1:nrow(LifeTable)){if (LifeTable[ciclo,15]==0) LifeTable[ciclo,15] <-
minimLarvae;
                    if (LifeTable[ciclo,16]==0) LifeTable[ciclo,16] <-
minimNymphs;
                    if (LifeTable[ciclo,17]==0) LifeTable[ciclo,17] <- minimAdults}

container2 <- matrix(data=0, nc=8, nr=8, byrow=TRUE)
for (ciclo in 1:nrow(LifeTable))
{
  container<-matrix(0, nr=8, nc=8, byrow=TRUE)
  container[1,8]<-2000
  container[2,1]<-LifeTable[ciclo,1]
  container[2,2]<-LifeTable[ciclo,5]
  container[3,2]<-LifeTable[ciclo,2]
  container[3,3]<-LifeTable[ciclo,6]
  container[5,4]<-LifeTable[ciclo,3]
  container[5,5]<-LifeTable[ciclo,7]
  container[7,6]<-LifeTable[ciclo,4]
  container[7,7]<-LifeTable[ciclo,8]
  container[4,3] <- LifeTable[ciclo,9]
  container[4,4] <- LifeTable[ciclo,12]
  container[6,5] <- LifeTable[ciclo,10]
  container[6,6] <- LifeTable[ciclo,13]
  container[8,7] <- LifeTable[ciclo,11]
  container[8,8] <- LifeTable[ciclo,14]
}
}
setwd("~Desktop/assessment_Boophilus/")
```

```
write.csv(ClimateTemp, file = "Temperaturas mensuales.csv",
row.names=FALSE,quote = FALSE,eol = "\n")
write.csv(ClimateVap, file = "Vapores mensuales.csv", row.names=FALSE,quote =
FALSE,eol = "\n")
write.csv(ClimatePre, file = "Precipitaciones mensuales.csv",
row.names=FALSE,quote = FALSE,eol = "\n")
write.csv(ClimateDS, file = "Deficits mensuales.csv", row.names=FALSE,quote =
FALSE,eol = "\n")
write.csv(patchesTable, file = "Datos de ciclo.csv", row.names=FALSE,quote =
FALSE,eol = "\n")
```
